# Genetic Variants in the Extracellular Matrix Gene *TNXB* Predicted to Alter Fibronectin III Domains in Arterial Aneurysmal and Dissection Diseases

**DOI:** 10.3390/ijms26136535

**Published:** 2025-07-07

**Authors:** Charlene Norgan Radler, Tianci Wang, Jaden LeGate, Lily Crone, Parminder Deo, Jacob Wortley, Peyton Moore, Griffin Bryant, Katherine Smitherman, Mohanakrishnan Sathyamoorthy

**Affiliations:** 1 Sathyamoorthy Laboratory, Department of Medicine, Anne Burnett Marion School of Medicine at TCU, Fort Worth, TX 76104, USA; 2 College of Arts and Sciences, Cornell University, Ithaca, NY 14850, USA; 3 Consultants in Cardiovascular Medicine and Science, Fort Worth, TX 76104, USA; 4 Fort Worth Institute for Molecular Medicine and Genomics Research, Fort Worth, TX 76104, USA

**Keywords:** *TNXB*, aortic aneurysm, carotid aneurysm, splenic aneurysm, arteriovenous malformation, bicuspid aortic valve, extracellular matrix, collagen matrix protein, fibronectin, genetic, single nucleotide variant, frameshift variant, AlphaFold

## Abstract

Arterial aneurysms are vascular conditions associated with life-threatening consequences in patients, such as dissection and rupture. Understanding their genetic basis is an evolving field, driven by the robust reporting of genetic variants associated with aneurysms in patients. In this study, we present clinical and genetic data from nine unrelated subjects with arterial aneurysms who were identified to harbor rare variants in the *TNXB* gene, mainly affecting fibronectin type III (FNIII) domains. The cohort included three female and six male subjects with a mean age of 53.5 years (SD = 14.4). The most frequently affected vascular territory was the thoracic ascending aorta (*n* = 7). A range of pathogenic impacts was predicted via multiple in silico tools that analyze evolutionary conservation and biochemical properties. Computational protein structure modeling with AlphaFold 3 predicted domain-specific alterations across multiple FNIII regions for four unique missense variants and one in-frame deletion, and premature protein truncation resulting from two frameshift variants. To our knowledge, this study is one of the first and largest to associate *TNXB* variants with arterial aneurysmal disease. Our findings demonstrate the potential of computational genomics and structural modeling to advance the understanding of extracellular matrix gene alterations in aneurysm pathogenesis.

## 1. Introduction

Arterial aneurysms are pathological dilatations of blood vessels involving all three layers of the arterial wall. While they can develop throughout the arterial tree, their prevalence and risk vary by anatomical location and are further influenced by factors such as gender and age [1]. A recent cohort study suggests that among patients 18 to 60 years of age, women are more likely to develop medium-vessel aneurysms (e.g., carotid/cerebral), while men more commonly present with large-vessel aneurysms (e.g., thoracic and abdominal aortic) [1]. In both genders, the abdominal aorta remains the most affected site of aortic aneurysm development, followed by the thoracic aorta, with an estimated prevalence of 5 to 10 per 100,000 person years [2,3]. Within the thoracic aorta, the root and ascending portions account for around 60% of the cases, followed by the descending aorta (30%) and the aortic arch (<10%) [3]. Less common sites of aneurysm development include the coronary arteries, intracranial arteries, and external iliac artery [3].

Many aneurysms, particularly aortic aneurysms, remain clinically silent until deleterious complications such as dissection or rupture occur [4]. As aneurysms enlarge, patients may develop symptoms based on anatomical location and adjacent structure involvement, such as dysphagia, low back pain, or cardiac murmurs if involving the aortic valve [4].

The genetic influence of aneurysmal diseases has been heavily researched, especially in TAAs, where up to 20% of affected individuals have a first-degree relative with the condition [5,6]. This figure is likely underestimated due to the lack of routine imaging in family members [6]. For many seemingly isolated TAA cases, the proband may be the first in a potential line of inheritance [7]. Despite advances in identifying genetic risk factors and increasing the use of genetic testing in suspected cases, further research is needed to refine risk stratification and improve diagnostic capabilities.

Thoracic aortic aneurysms are further classified into three categories: syndromic, familial non-syndromic, or sporadic [8]. Syndromic TAAs, responsible for roughly 20% of the cases, are aneurysms that occur in association with systemic diseases such as Marfan Syndrome, Ehlers–Danlos Syndrome (EDS), or Loeys–Dietz Syndrome [8]. In contrast, familial non-syndromic aneurysms occur without other systemic abnormalities and demonstrate a family inheritance pattern typically acquired in an autosomal dominant fashion [8]. Lastly, sporadic TAAs occur without systemic disease or familial pattern, which can result from de novo gene variants or secondary to damage to the aorta associated with trauma, hypertension, or aging [9]. Currently, 11 genes have been established as high-risk for TAA formation, which include *ACTA2*, *MYLK*, *COL3A1*, *PRKG1*, *SMAD3*, *FBN1*, *MYH11*, *TGFβR1*, *TGFβ2*, *LOX*, and *TGFβR2* [10]. Variants in these genes impact vascular smooth muscle function and/or metabolism, extracellular matrix (ECM) integrity, or transforming growth factor ß (TGF-β) formation and signaling pathways [10].

**Figure 1:**
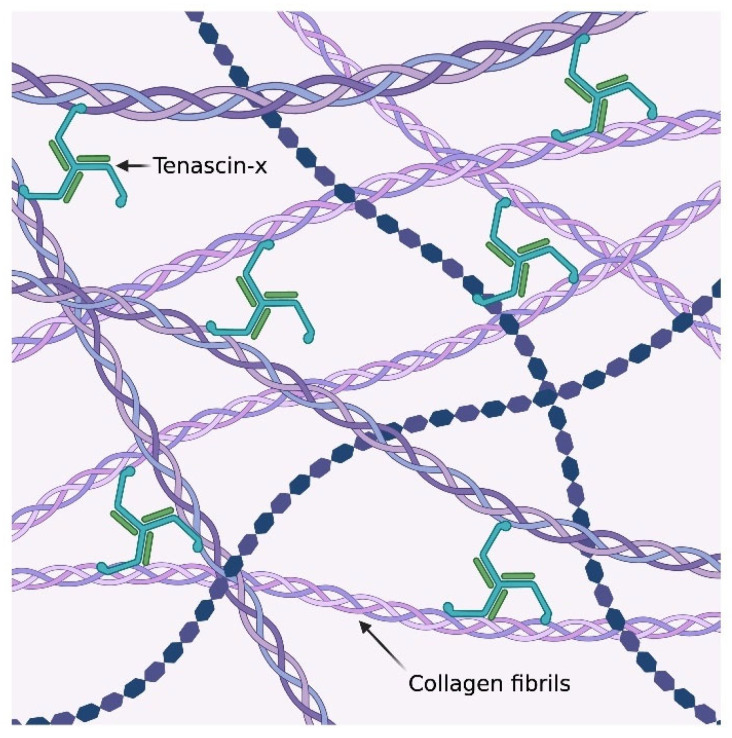
The trimeric structure of tenascin-x and its local interactions with the collagen fibrils within the extracellular matrix [15].

Among the genes implicated in arterial aneurysms, the potential role of tenascin XB (*TNXB*) remains largely underexplored. The *TNXB* gene, located on chromosome 6p21.3, encodes the protein tenascin-X (TNX), a large ECM glycoprotein widely expressed in various organs such as the heart, lungs, adrenal glands, and skin [11]. Structurally, TNX assembles as a disulfide-linked trimer (Figure 1) tethered at the N-terminus and contains multiple functional domains, including 32 fibronectin type III (FNIII) repeats [11]. These multimodular domains are key to TNX roles in collagen fibrillogenesis, cell adhesion, and the regulation of TGF-β signaling [11,12].

Variants in *TNXB* have been linked to classic-like Ehlers–Danlos Syndrome (cEDS) and are characterized by skin hyperextensibility and joint flexibility without atrophic scarring or wound-healing defects [13]. Additionally, *TNXB* haploinsufficiency has been identified in individuals with incomplete penetrance and is characterized by chronic musculoskeletal pain and hypermobile EDS [14]. In this study, we describe the clinical and genetic profiles of nine unrelated patients with arterial aneurysms harboring rare *TNXB* variants, most of which affect FNIII domains. Additionally, using AlphaFold 3 and multiple in silico prediction tools, we model the potential structural and functional consequences of these variants.

## 2. Results

### 2.1. Clinical Features

Clinical genetic testing was offered in all cases of non-syndromic and sporadic arterial aneurysms to assess the presence of high-risk, pathogenic variants that could influence long-term clinical surveillance and management. Of the 170 individuals who underwent clinical genetic screening in our program following the discovery of aneurysms, 9 unrelated patients (*N* = 9) who were found to have genetic variants in *TNXB* and arterial aneurysms (Table 1) were included in the study cohort. No other pathogenic variants, VUS, gross deletions, or gross duplications were identified in the 34 additional genes included in the screening panel.

Our study cohort consists of three females and six males with a mean age of 53.5 years (SD = 14.4) at the time of diagnosis. Aneurysm locations varied: 78% had TAAs and 22% had aneurysms in other arterial beds (e.g., carotid, cerebral, splenic, or iliac). All TAAs were in the ascending aorta and ranged in size from 42 mm to >55 mm. Aneurysm dissection was reported in one subject.

Clinical presentation also varied across the cohort: 67% were diagnosed incidentally during imaging (e.g., CT angiography or TTE) for unrelated reasons or for standard-of-care symptom-driven evaluation. A history of hypertension was present in 8 of 9 subjects, and significant aortic insufficiency was found in 3 of 9 subjects. All subjects had normal ejection fractions (EF > 60%).

Three additional *TNXB* variants were identified in two individuals not included in this study cohort: c.3149-3C>T in a patient with cEDS and Q961R/V882M in a patient with a family history of multiple aortic dissections. Neither patient has an aneurysmal phenotype, and both continue to undergo clinical surveillance.

### 2.2. Genetic Results

**Figure 2:**
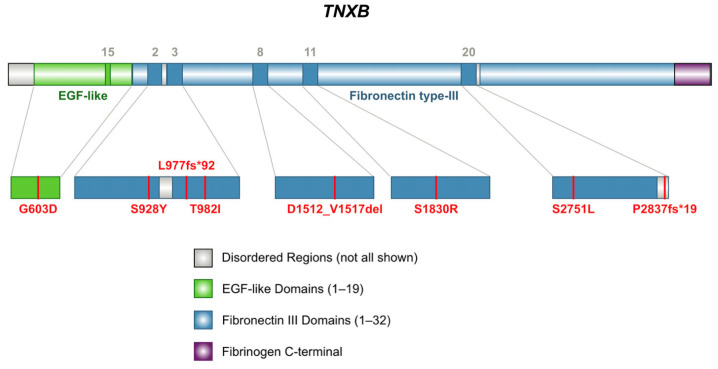
Schematic representation of domain locations of *TNXB* variants found in nine subjects with arterial aneurysms. Two subjects carry the same variant, S1830R [17].

*TNXB* variants identified in our cohort include two pathogenic frameshift variants predicted to result in premature protein products, one six-amino acid in-frame deletion, and six missense variants, see Figure 2. Four variants are classified as variants of uncertain significance (VUS), which the American College of Medical Genetics and Genomics (ACMG) defines as one that cannot be definitively classified as either pathogenic or benign, prompting further investigation to clarify its role in disease [16]. Two variants have conflicting interpretations of pathogenicity (Table S1), which reflects multiple classifications among submissions using the ACMG standards [16].

All the variants had overall allele frequencies of less than 0.1% reported in gnomAD, indicating that they are rarely found in the population, see Table S1 [18]. The results from computational modeling illustrated in the following sections demonstrate predicted alterations in the overall protein structure for L977fs*92 and P2837fs*19 and predicted impacts to fibronectin-type III (FNIII) domains arising from the missense variants. Finally, we describe patterns of pathogenicity resulting from in silico analyses for multiple missense variants.

#### 2.2.1. Frameshift Variants

Two subjects carry pathogenic frameshift variants, p.L977fs*92 (c.2929delC) [19] and p.P2837fs*19 (c.8510_8511delCT) [20]. These variants are predicted to disrupt protein function by either nonsense-mediated mRNA decay or truncation, resulting in premature protein products [19,20]. Each variant has a single submission in ClinVar associated with a cardiovascular phenotype.

**Figure 3:**
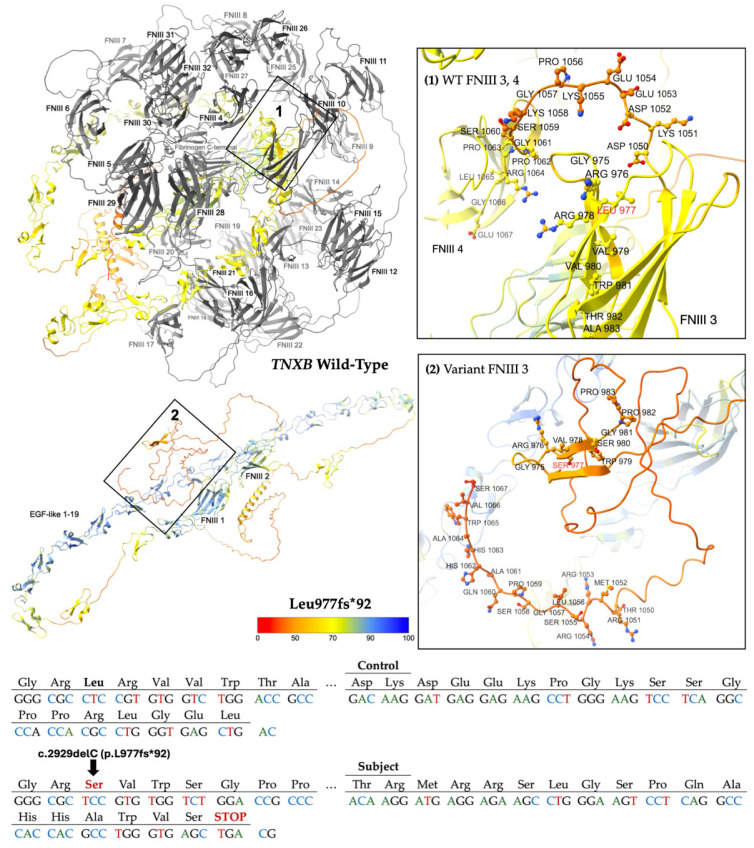
Modeling comparing wild-type (WT) *TNXB* [top left] to the truncated protein product L977fs*92 [bottom left]. The WT domains FNIII 3 to 32 and fibrinogen-like c-terminal [top left], which are predicted to be deleted, are colored gray. Otherwise, structures are colored by per-atom pLDDT, and a key is depicted. Inset boxes 1 and 2 magnify the protein chain and amino acids nearby WT Leu 977 [top right] and variant Ser 977 [bottom right], respectively. WT and variant structures are computationally predicted using the AlphaFold 3 (AF3) server [21]. Depicted at the bottom of the figure is the abbreviated mutated sequence of 92 amino acids with the control sequence provided for comparison. A diagram of the full mutated sequence is available as Scheme S1.

L977fs*92 results from the deletion of one nucleotide at position 2929 of NM_001365276.2 (hg38, chr6: 32085969), inducing a translational frameshift with a predicted alternate stop codon 92 residues downstream. This allele has a reported frequency of 0.0003109% (5/1608118) in gnomAD, suggesting it is a very rare variant in the population [18]. Computational modeling using AlphaFold 3 reveals a protein structure (Figure 3) missing 30 of the 32 fibronectin-type III (FNIII) domains and the fibrinogen-like c-terminal, which are crucial to *TNXB* function and ECM integrity [21].

**Figure 4:**
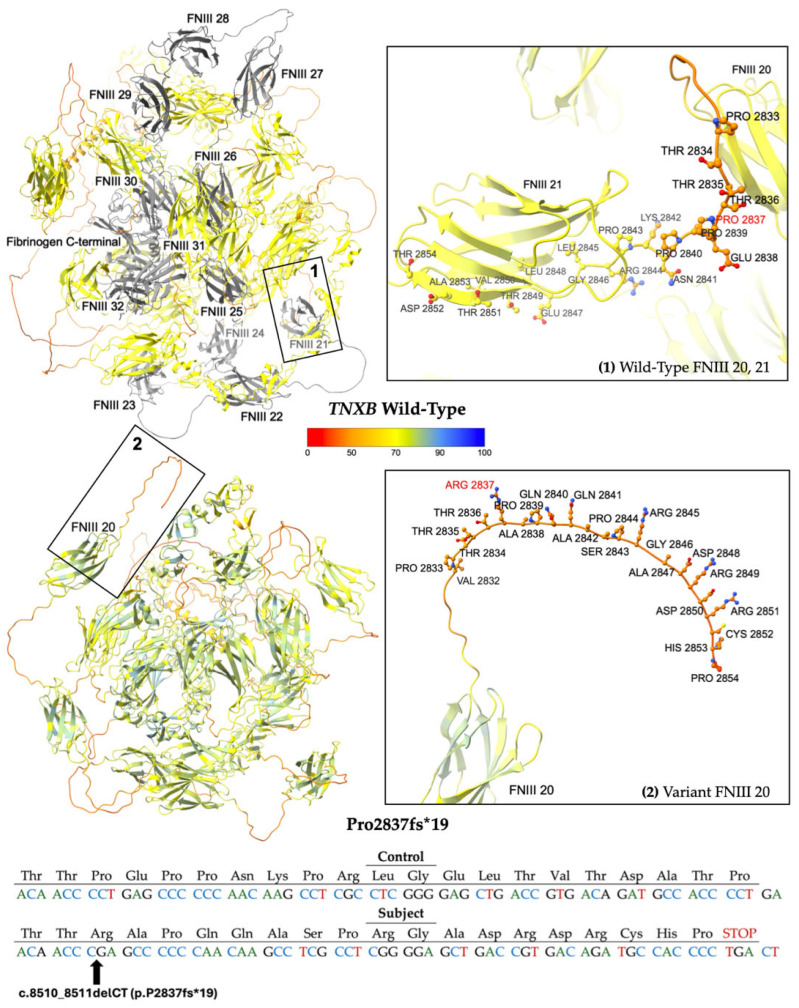
Modeling comparing wild-type (WT) *TNXB* [top left] to the truncated protein product P2837fs*19 [bottom left]. The WT domains FNIII 21 to 32 and fibrinogen-like c-terminal [top left], which are predicted to be deleted, are colored gray. Otherwise, structures are colored by per-atom pLDDT, and a key is depicted. Inset boxes 1 and 2 magnify the protein chain and amino acids nearby WT Pro 2837 [top right] and variant Arg 2837 [bottom right], respectively. WT and variant structures are computationally predicted using the AF3 server [21]. Depicted at the bottom of the figure is the mutated sequence of 19 amino acids with the control sequence provided for comparison.

The second frameshift variant P2837fs*19 results from the deletion of two nucleotides at positions 8510 to 8511 of NM_019105.8 (hg38, chr6: 32053662–32053663), leading to a translational frameshift and a predicted premature stop codon 19 residues downstream (Figure 4). The allele has a reported frequency of 0.00006199% (1/1613288) in gnomAD, suggesting it is a very rare variant in the population [18]. Computational modeling using AlphaFold 3 reveals a protein structure (Figure 4) missing FNIII domains 21 through 32 and the fibrinogen-like c-terminal [21].

#### 2.2.2. In-Frame Deletion Variant

A heterozygous variant of uncertain significance, p.D1512_V1517del, was isolated in *TNXB* coding Exon 11. The variant results from an in-frame deletion, c.4535_4552del18, at nucleotide positions 4535 to 4552 of NM_001365276.2 (hg38, chr6: 32073776–32073793). The allele has an overall frequency of 0.01141% (184/1612820) as reported by gnomAD, suggesting it is not a common variation in the population [18]. The highest observed frequency is 0.01492% (176/1179598) among European (non-Finnish) alleles [18].

**Figure 5:**
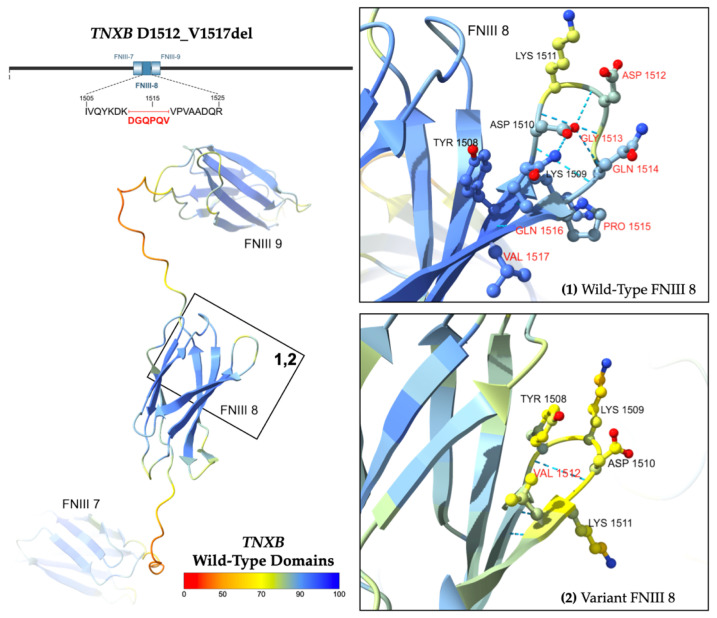
Predicted structure of WT *TNXB* FNIII domains 7 to 9 [left] and D1512_V1517del. Inset boxes 1 and 2 [right] magnify the protein chain and amino acids near the variant. Amino acids affected by the variant are labeled in red. Structures are colored by per-atom pLDDT, and a key is depicted. The WT structure was obtained from the AlphaFold Database (AFDB) [22,23]. The variant structure was computationally predicted using the AF3 server [21].

Computational modeling in AlphaFold 3 demonstrates the predicted protein structure (Figure 5) [21]. The wild-type domain is illustrated as a beta-sandwich fold comprising seven antiparallel beta strands linked by hairpin turns. The in-frame deletion of six amino acids (DGQPQV) from codons 1512 to 1517 is predicted to truncate a hairpin turn and beta strand within the eighth fibronectin type-III domain.

#### 2.2.3. Missense Variants

Five unique heterozygous missense variants were identified (Figure 2). The variant G603D (Subject 4) is located in EGF-like domain 15, while all the other missense variants are located within FNIII regions. Three missense variants were classified as variants of uncertain significance (VUS), and two were classified with conflicting reports of pathogenicity based on aggregate records in ClinVar, see Table S1 [24]. The conditions associated with these variants include Ehlers–Danlos Syndrome and vesicoureteral reflux (VUR) [24]. The variant S1830R was also identified in children with VUR and joint hypermobility [25].

**Figure 6:**
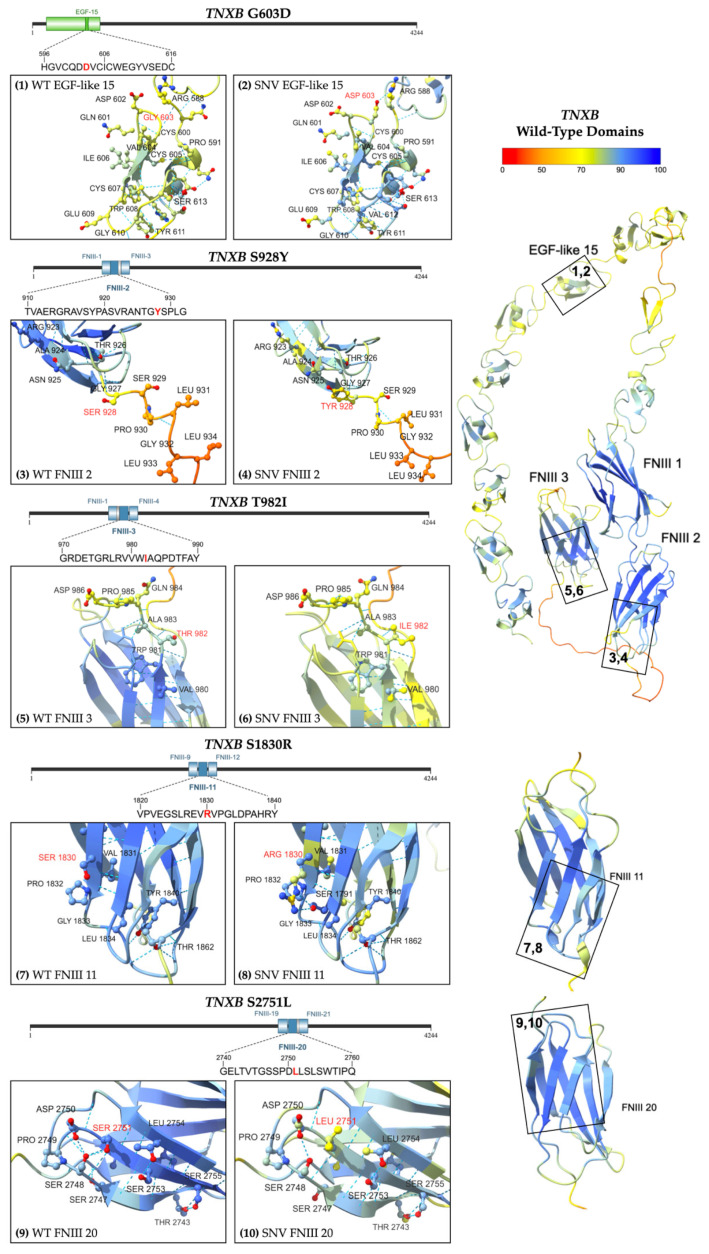
Predicted structure of WT *TNXB* FNIII and EGF-like domains [right] with numbered boxes indicating the location of each missense variant. Inset boxes 1 and 2 [upper left row 1] magnify the protein chain and amino acids near (1) WT Gly 603 and (2) variant Asp 603. Inset boxes 3 and 4 [upper left row 2] magnify (3) WT Ser 928 and (4) variant Tyr 928, respectively. Inset boxes 5 and 6 [middle left row 3] magnify (5) WT Thr 982 and (6) variant Ile 982, respectively. Inset boxes 7 and 8 [bottom left row 4] magnify (7) WT Ser 1830 and (8) variant Arg 1830. Inset boxes 9 and 10 [bottom left row 5] magnify (9) WT Ser 2751 and (10) variant Leu 2751. Models are colored by per-atom pLDDT, and a key is depicted. The WT structure was obtained from AFDB [22,23], and the variant structure was computationally predicted using the AF3 server [21].

All the variants had overall allele frequencies of less than 0.1% reported by gnomAD, indicating they are rarely found in the population [18]. Two variants were found to be much more prevalent in certain genetic ancestry groups than in the general population. The highest observed frequency of T982I was 0.4467% (335/75,000) among African/African American alleles, and S1830R was most often observed in the Ashkenazi Jewish population with a frequency of 0.3078% (91/29,566) [18]. Subjects 7 and 8 share the same variant (S1830R) in FNIII domain 11, while a more distal domain is affected in Subject 9 (S2751L, FNIII domain 20). Predictive modeling of the protein structures was performed using AlphaFold 3 (Figure 6) [21].

In silico analysis was performed using an array of well-validated tools for gene discovery research. The results predicted a range of possible pathogenic variant impacts arising from evolutionary conservation and changes in structure and physicochemical properties, see Table 2. Clear patterns of pathogenicity were established for multiple variants, and conflicting results are reported. Notably, variants G603D, S928Y, T982I, and S2751L were predicted to be pathogenic by multiple tools. Most variants, except S1830R, were predicted to be in conserved regions (GERP++, PhyloP, and Align GVGD) [26,27,28,29] and thus may be more likely to cause deleterious effects. Furthermore, variants G603D and S2751L were predicted to have damaging impacts by most tools utilizing conservation data, including SIFT [30]. In contrast, few tools (FATHMM v2.3 and PrimateAI) [31,32], which analyze evolutionary conservation, predicted the functional impact of most variants to be tolerated.

Variant G603D was predicted to be pathogenic by most tools utilizing supervised machine learning algorithms (BayesDel, CADD v1.7, and VEST4) [33,34,35]. Four missense variants were predicted by CADD v1.7 [34] to be among the top 1% most deleterious variants, although most other tools that utilize supervised machine learning algorithms, including REVEL [36], predicted each missense variant to be tolerated. Finally, MutPred2 and PolyPhen-2 v2.2 [37,38] both predicted G603D and S2751L to deleteriously impact protein sequence or structure. MutPred2 predicted a 40% likelihood that S2751L leads to loss of intrinsic disorder (*p* = 0.02). Additionally, MutPred2 predicted multiple molecular effects from G603D, including a 25% probability of altered metal binding (*p* = 0.03), a 13% likelihood of altered transmembrane protein (*p* = 0.02), a 13% likelihood of loss of disulfide linkage at C605 (*p* = 0.04), and a 9% probability of gain of pyrrolidone carboxylic acid at Q601 *(p* = 0.01).

## 3. Discussion

Identifying gene variants associated with arterial aneurysms is essential to understanding their pathogenesis and improving diagnosis and risk stratification for life-threatening complications such as thoracic aortic dissection. Numerous genetic contributors have been identified to date, including genes that influence cytoskeletal integrity, smooth muscle contraction, TGF-β signaling, and ECM proteins such as collagen, lysyl hydroxylase, and fibrillin [10,39]. This study provides novel insights into this body of research by reporting variants in *TNXB*, an ECM gene not well characterized in this context, among a cohort of nine individuals with arterial aneurysms.

*TNXB* encodes Tenascin-X (TNX), the largest member of the tenascin family of ECM glycoproteins [40]. The globular protein structure of TNX includes a hydrophobic domain with three heptad repeats, multiple EGF-like repeats, thirty-two FNIII repeats, an RGD (arginine–glycine–aspartic acid) cell recognition site between the ninth and tenth FNIII repeats, and a C-terminal fibrinogen-like domain (FBG) [40]. TNX assembles into a disulfide-linked trimer via its N-terminal domains and binds directly to collagen fibrils to help organize them [11,41]. TNX also regulates cell adhesion during physiologic processes, including endothelial-to-mesenchymal transition via integrin-mediated cell attachment primarily at its FBG domain [42,43]. Uniquely, *TNXB* overlaps the *CYP21A2* gene encoding steroid 21-hydroxylase at its 3′ end [40].

TNX is widely expressed in tissues throughout the body and is predominantly found in the heart and muscle [13,44]. TNX is initially expressed during embryogenesis by epicardial cells near the AV groove and atrium, eventually migrating all over the heart, including within developing coronary vasculature [44,45]. The protein’s widespread tissue expression, complex domain structures, and distinct genomic positioning all contribute to its importance in maintaining ECM integrity and regulating signaling pathways. TNX dysfunction has been associated with a range of clinical phenotypes, including connective tissue disorders, myopathies, vesicoureteral reflux, adrenal hyperplasia, neurological syndromes, and, based on our findings, arterial aneurysms [46,47].

While many *TNXB* variants are linked to cardiovascular phenotypes in ClinVar, their clinical characterization is not well known. Most subjects in our study cohort were asymptomatic at presentation, with aneurysms detected incidentally on imaging. This presentation pattern is consistent with the often-silent progression of aneurysms and underscores the need for vigilant clinical surveillance in patients with *TNXB* variants. Additionally, Subject 4’s phenotype of severe aortic insufficiency secondary to bicuspid aortic valve (BAV) at the age of 40 years with development of an ascending aortic aneurysm following aortic valve replacement is comparable to existing studies. In a recent abstract, authors observed a 10-fold higher prevalence of BAV than the general population in three subjects of a cohort (*N* = 19) containing two or more *TNXB* variants [48]. While BAV is recognized to increase the risk of aortic aneurysm development and is frequently associated with genetic variants [49,50], it is unclear whether *TNXB* variants are associated with differences in clinical severity or outcomes among individuals with nonsyndromic BAV due to the lack of widespread inclusion on heritable thoracic aortic disease panels.

Many of our patients presented with ascending thoracic aortic aneurysms, despite the abdominal aorta being the most common site for aneurysms in the general population. Additionally, two individuals exhibited less common splenic and internal carotid aneurysms. This distribution contrasts with prior studies, including those by Korfer et al., where a heterozygous *TNXB* variant was identified in a patient with five arterial aneurysms spanning from the abdominal aorta to the popliteal artery [51]. A more recent abstract by Neogi et al. observed a statistically significant association between carrying two or more *TNXB* variants and aneurysms in multiple vascular beds, but specific aneurysm locations were not detailed [48]. Given the role of *TNXB* in vascular ECM integrity and its ubiquitous expression across various tissues, further investigation is needed to determine whether *TNXB* alterations demonstrate a predilection towards the thoracic ascending aorta or impact the entire arterial tree. Additionally, conflicting findings from prior studies comparing TNX expression levels in aneurysmal tissue samples compared with healthy tissue [52,53] highlight the need for further tissue-level investigation into the possible mechanistic contributions of *TNXB* to aneurysm formation.

This current study identifies two frameshift variants, L977fs92 and 2837fs19, predicted to result in the loss of the fibrinogen C-terminal, numerous FNIII domains, and the RGD recognition site. Additionally, this study presents six missense variants predicted to impact multiple FNIII domains and one EGF-like domain. Here we review the pathogeneses of known disorders related to changes in *TNXB* structure and function and discuss potential biomolecular mechanisms that may contribute to arterial aneurysm formation.

Nearly 70% of the *TNXB* frameshift variants in ClinVar (*n* = 71) are associated with EDS, which is well-described in the literature [24]. Complete deficiency of *TNXB* causes an autosomal recessive form of classical-like EDS (cEDS) characterized by joint hypermobility, hyperextensible skin, and easy bruising without atrophic scars [54,55,56]. A heterozygous loss of function causing *TNXB* haploinsufficiency leads to a milder hypermobility form of cEDS that lacks skin features and was first observed in family members of individuals with cEDS [14]. *TNXB* haploinsufficiency has also been documented among a cohort of CAH patients with a 21-hydroxylase deficiency due to the gene’s 3′ overlap with *CYP21A2*, a condition known as CAH-X syndrome [57]. Six of thirteen probands had structural heart abnormalities, including valvular abnormalities, ventricular enlargement, and a congenital ventricular diverticulum [57]. Further, one proband had a heterozygous *TNXB* frameshift mutation associated with joint hypermobility and cardiac chamber enlargement [57]. Of note, neither of the subjects with frameshift variants in our cohort exhibited CAH-X phenotype, despite their variants resulting in the complete loss of the C terminus.

Several mechanisms may explain how *TNXB* mutations contribute to aneurysm formation, including fibrillogenesis, cell signaling, and endothelial-to-mesenchymal (EndMT) transition [13]. Fibrillogenesis involves the assembly of ECM proteins, such as collagen and elastin, which is critical for vascular integrity and is frequently disrupted in thoracic aortic aneurysms [58]. TNX interacts with type I and VI collagens and increased the rate and quantity of collagen fibril formation during several in vitro experiments [41,59]. Additionally, skin tissue from *TNXB*-deficient mice demonstrates reduced collagen density in the ECM [60]. However, whether TNX exerts a regulatory role during collagen fibrillogenesis remains an active question. One study demonstrated recombinant TNX increased collagen gel stiffness without affecting collagen fibrillogenesis, possibly by strengthening molecular interactions between fibrils [61].

*TNXB* deficiency has been linked to alterations in endothelial TGF-β signaling during endothelial-to-mesenchymal (EndMT) transition in human endothelial and mouse aorta cells [42,62]. These processes are implicated in inflammatory stress, endothelial dysfunction, and subsequent aneurysm development [42]. The dedifferentiation of vascular smooth muscle cells is counter-regulated by the TGF-β promotion of contractile proteins [62], and in prior in vitro experiments, TNX appears to use its fibrinogen-like (FBG) domain to bind directly to TGF-β [42,43]. Furthermore, one study suggests that FNIII and FBG domains utilize distinct signaling pathways to regulate EndMT [11,43]. We speculate that TNX variants could alter TGF-β activation and reduce collagen fiber density, leading to dysregulated cell plasticity and weakened vascular walls in the formation of arterial aneurysms.

Multiple FNIII domains, including 2, 3, and 11, were impacted by variants within our cohort, suggesting certain regions may have increased susceptibility to mutations. TNX FNIII domains are around 100 amino acids in length and adopt a characteristic beta-sandwich configuration with seven interconnected β-strands, accounting for a large portion of the protein’s 4244 amino acids [41,63]. The FNIII domain reversibly folds and unfolds, a feature attributable to its considerable domain-level variability in stability, numerous inter-strand loops, and the absence of internal, stabilizing disulfide bonds [41,63]. The flexibility and elasticity of FNIII domains permit a broad range of domain conformations, supporting their diverse roles in fibrillogenesis, wound healing, and mechanotransduction [11,13].

FNIII domains are also found in several other cardiovascular proteins, particularly myosin light chain kinase (*MYLK*), myosin-binding protein C, and titin (*TTN*). An *MYLK* mutation has been linked to thoracic aortic aneurysm and dissections (TAAD) in a recent case by Boelman et al., where a pathogenic splice variant that disrupted the FNIII domain was associated with thoracic aortic disease in the proband and his mother [64]. In addition, pathogenic variants affecting the FNIII domain in *TTN* have been strongly implicated in structural myocardial diseases [65,66]. Given the functional roles of FNIII domains and their known involvement in the pathologies of other cardiovascular proteins, altered folding or stability in FNIII domains may offer a plausible mechanism for the phenotypes observed in our patients.

This study is among the *first* and *largest* to report on the association between aneurysmal disease and genetic variants in *TNXB.* Our biocomputational analyses predicted alterations in protein structure that may explain the observed development of arterial aneurysmal diseases in humans. Based on the combined evidence, from image-confirmed phenotypes to in silico modeling, we recommend further mechanistic studies of *TNXB* variants. Incorporating genetic screening for genes such as *TNXB* into clinical care for patients with newly diagnosed aneurysms could help clarify the prevalence and impact of these variants and identify patients at risk for future aneurysm development. Although this initial report is limited in sample size and will be expanded through ongoing surveillance in our program, it aims to promote further mechanistic research behind *TNXB* and its potential association with arterial aneurysm development through clinical investigation in larger cohorts. Ultimately, this work may warrant re-designating these variants in *TNXB* as either very likely pathogenic or pathogenic by ACMG criteria.

## 4. Materials and Methods

### 4.1. Next-Generation Sequencing Analysis

A commercial panel (TAADNext by Ambry Genetics, Aliso Viejo, California, USA) consisting of 35 genes associated with extracellular matrix disorders was utilized to assess for genetic variants in patients with aneurysmal disorders in our program using their respective National Center for Biotechnology Information (NCBI) reference sequences [67]. The list of 35 genes and their respective NCBI sequences are listed in Table S2.

To identify variants, next-generation sequencing was conducted in coding domains, untranslated regions, and intronic domains. The NCBI published sequence for *TNXB* was used for analysis of the gene. Various methods, including gross deletion and gross duplication, were investigated to determine the gene copy number for covered exons and untranslated regions of the genes in Table S2. Bait capture was used to enrich coding exon sequences via “biotinylated oligonucleotide probes and subsequent polymerase chain reaction and sequencing and utilizing NCBI reference sequences [67]. Sanger sequencing was additionally used for those regions that were missing or for those with poor and insufficient read depth coverage for reliable detection of variants”. Standard clinical genetic counseling was provided to each patient.

### 4.2. Variant Analysis and Computational Prediction

We performed a comprehensive analysis of the potential impact of each variant, including population frequency, clinical data, in silico analysis, and computational protein modeling. Our methodology utilized publicly available databases and tools commonly utilized for gene discovery research. We gathered protein functional data from the Uniprot database [68], aggregate variant-level data from the ClinVar database [24], and population frequencies from the Genome Aggregation Database (gnomAD) [18]. Clinical data, including disease presentation, past medical history, and aneurysm features, was obtained from retrospective chart review.

We performed in silico analysis for the missense variants using an array of well-validated prediction tools to characterize the potential impact of each variant, including evolutionary conservation and structural/physicochemical parameters [69]. Tools including Align GVGD (http://agvgd.hci.utah.edu, accessed on 15 May 2025, Huntsman Cancer Institute, Salt Lake City, Utah, USA), FATHMM v2.3 (University of Bristol, Bristol, UK), GERP++ (Stanford University, Stanford, CA, USA), PhyloP (Cold Spring Harbor Laboratory, Cold Spring Harbor, New York, NY, USA), PrimateAI v0.2 (Illumina Inc, San Diego, CA, USA), and SIFT (https://sift.bii.a-star.edu.sg, accessed on 15 May 2025, Genome Institute of Singapore, Singapore) [26,27,28,29,30,31,32] analyzed how each variant altered a conserved region, while MutPred2 (Indiana University, Bloomington, IN, USA) and PolyPhen-2 v2.2 (Harvard University, Cambridge, MA, USA) [37,38] analyzed potential impact on protein sequence or structure. Finally, BayesDel (https://fenglab.chpc.utah.edu/BayesDel.html, accessed on 15 May 2025, The University of Utah, Salt Lake City, UT, USA), CADD v1.7 (University of Washington, Seattle, WA, USA), REVEL (https://sites.google.com/site/revelgenomics/, accessed on 15 May 2025, Stanford University, Stanford, CA, USA), and VEST4 (Johns Hopkins University, Baltimore, MD, USA) [33,34,35,36] utilized supervised machine learning algorithms to predict variant pathogenicity. Scores were calculated utilizing prediction tool platforms except for BayesDel, GERP++, PhyloP, and REVEL, which were obtained from the UCSC Genome Browser (https://genome.ucsc.edu/, accessed on 27 December 2024, University of California Santa Cruz, Santa Cruz, CA, USA) [70]. The scores were interpreted according to output and thresholds provided by their respective platforms for gene discovery research, which differ from recommendations for clinical variant classification by Pejaver et al. [71].

We utilized the AlphaFold database (AFDB) [22,23] and AlphaFold3 (AF3) server [21] to obtain predicted structures based on AlphaFold’s validated atomic-level accuracy and ease of use. The predicted structure of the wild-type *TNXB* protein was extracted from the whole proteome download in the AFDB as overlapping fragments of 1400 amino acids. The selected *TNXB* fragments for each missense and in-frame deletion variant are fragment 2 (G603D, S928Y, and T982I), fragment 5 (D1512_1517del), fragment 6 (S1830R), and fragment 11 (S2751L). The corresponding FASTA sequence was modified for each wild-type fragment to reflect the amino acid change for the missense and in-frame deletion variants.

We modeled the full wild-type protein for *TNXB* using the NCBI reference sequences associated with each frameshift variant. The coding sequence for *TNXB* transcript variant XB (NM_019105.8) [72] was modified to reflect the two base-pair deletion c.8510_8511delCT and translated to the corresponding protein sequence for P2837fs. The coding sequence for *TNXB* transcript variant 3 (NM_001365276.2) [73] was modified to reflect the one-base pair deletion c.2929delC and translated to the corresponding protein sequence for L977fs. The hg38 genome assembly was utilized for all the protein sequences.

Structure predictions were obtained from the modified sequences for each variant using the AlphaFold 3 server, and top-ranked structures were selected from multiple predictions. AlphaFold structure predictions are freely available for both academic and commercial use under Creative Commons Attribution 4.0 (CC-BY 4.0) license terms [21]. Molecular graphics and structure analyses were performed using UCSF ChimeraX (https://www.rbvi.ucsf.edu/chimerax accessed on 27 December 2024), developed by the Resource for Biocomputing, Visualization, and Informatics at the University of California, San Francisco, with support from National Institutes of Health R01-GM129325 and the Office of Cyber Infrastructure and Computational Biology, National Institute of Allergy and Infectious Diseases [74]. The selected structures were colored by per-atom pLDDT, which is an estimate of confidence in a predicted structure agreeing with an experimental structure. The global pLDDT was near or greater than 70 for each structure, indicating a reliable backbone prediction, see Table S3 [22]. Higher pLDDT values, such as those colored in blue in Figure 3, Figure 4, Figure 5 and Figure 6, demonstrate higher confidence in the predicted structure.

Ethical review and study approval were obtained from Texas Christian University under IRB#2025-118. Informed consents are on file at CCMS-FW. The data presented in this study is available upon request from the corresponding author but is not publicly available due to patient privacy considerations.

## 5. Conclusions

Arterial aneurysms are vascular pathologies that can lead to serious, and even fatal, complications, including dissection and rupture. This report adds to the current literature by describing the association between variants in *TNXB* and aneurysm development and advances the understanding of how disruptions in extracellular matrix proteins and their regulators may lead to vascular aneurysms. While many experimental studies are yet to be performed, the significant alterations to the structure of *TNXB* as identified in biocomputational predictive models provide promising insights into the potential implications on aneurysm development. Future mechanistic research concerning larger clinical, translational, and transgenic animal model investigations will help elucidate the role of *TNXB* in aneurysm pathogenesis, and doing so will help anchor this gene in aneurysmal pathogenesis and advance diagnostic criteria to more effectively risk-stratify patients in this dangerous disease state.

## 6. Limitations

This novel report, while exciting, is limited by sample size, and we acknowledge that computational methodologies for genetic and protein structure prediction must ultimately be verified through rigorous in vitro and in vivo experimental confirmation. The accurate experimental prediction of protein structures at the multi-domain level remains a significant barrier to understanding the implications of the rapidly expanding collection of genetic variants, such as those identified in this study. While experimental methods remain the “gold standard” of protein structure, advances in computational structure prediction in the form of AlphaFold, among others, have drastically reduced both time and resource barriers while exhibiting atomic-level accuracy. AlphaFold 2 was found to have an all-atom accuracy of 1.5 Å rmsd_95_ (95th percentile 1.2–1.6 Å) during the 14th Critical Assessment of protein Structure Prediction (CASP14), which is impressive considering that the average carbon atom exhibits a width of 1.4 Å [75]. However, the use of AlphaFold and other computational methods to determine the effect of mutations is controversial, with recent studies highlighting difficulties in obtaining accurate representations of the stability and function of mutated proteins [76,77,78,79]. This study employs AlphaFold 3 to better understand the potential effects of *TNXB* genetic variants given the lack of multi-domain experimental *TNXB* structures in the Protein Data Bank. We recognize the limitations of using predicted mutant structures to illustrate gross structural differences and restrict our discussion to hypothesizing possible alterations to protein behavior based on these predictive illustrations.

## Figures and Tables

**Table 1:** Clinical and diagnostic imaging characteristics for nine unrelated subjects with arterial aneurysms and variants in the *TNXB* gene. Abbreviations: AI, aortic insufficiency; AVMs, arteriovenous malformations; AVR, aortic valve replacement; BAV, bicuspid aortic valve; EF, ejection fraction; HTN, hypertension; PMHx, past medical history.

Subject	Variant	Age Sex	PMHx	Clinical Features	CT Angiography	Transthoracic Echocardiogram
1	p.L977fs*92	48 M	HTN	Asymptomatic	45 mm ascending aortic aneurysm	Normal structure, function. EF > 60%
2	p.2837fs*19	61 F	Stroke, HTN	Dizziness, tinnitus	4.1 × 5.2 mm right paraclinoid internal carotid aneurysm with a 3.8 mm neck	EF > 60%
3	p.D1512_V1517Del	57 F	HTN	Asymptomatic	42 mm ascending aortic aneurysm	Normal structure, function. EF > 60%
4	p.G603D	40 M	BAV s/p AVR for severe AI	Asymptomatic	43 mm ascending aortic aneurysm	Mechanical AVR, EF > 60%
5	p.S928Y	72 M	Splenic aneurysm and diffuse AVMs, HTN	GI bleeds	Splenic aneurysm, diffuse AVMs	Normal structure, function.
						EF > 60%
6	p.T982I	58 F	HTN	Asymptomatic	44 mm ascending aortic aneurysm	Mild AI,
						EF > 60%
7	p.S1830R	90 M	HTN	Ascending aortic aneurysm repair and AVR	51 mm ascending aortic aneurysm	Severe AI
						EF> 60%
8	p.S1830R	64 M	HTN	Asymptomatic	43 mm fusiform ascending aortic aneurysm	Normal structure, function. EF > 60%
9	p.S2751L	53 M	Ascending aortic dissection (>55 mm) with severe AI, HTN	Symptomatic dissection	Outside historical data	Mechanical AVR, EF > 60%

**Table 2:** Comparison of the in silico results for missense variants from an array of prediction tools using output and thresholds provided for gene discovery research. Variants are listed by column, and outputs are listed by row. Cells are colored by predicted impact with shades of green predicting benign/tolerated and shades of red predicting pathogenic/deleterious.

Tool	c.1808G>A, p.G603D	c.2783C>A, p.S928Y	c.2945C>T, p.T982I	c.5488A>C, p.S1830R	c.8252C>T p.S2751L
Align GVGD	93.77 Grade: C65	143.11 Grade: C65	89.28 Grade: C65	109.21 Grade: C65	144.08 Grade: C65
BayesDel (MaxAF)	0.20017 deleterious	−0.455478 tolerated	−0.483146 tolerated	−0.517306 tolerated	−0.068 tolerated
CADD v1.7	26.4 1% most deleterious	23.0 1% most deleterious	24.2 1% most deleterious	11.57 10% most deleterious	25.5 1% most deleterious
Evolutionary Action (EA)	43.08 neutral	60.74 neutral	54.27 neutral	3.58 more neutral	96.08 deleterious
FATHMM	2.51 tolerated	0.35 tolerated	3.49 tolerated	0.42 tolerated	−0.04 tolerated
GERP++	4.26 constrained	4.03 constrained	1.98 constrained	−2.02 unconstrained	5.0 constrained
MutPred2	0.869 pathogenic	0.269 benign	0.431 benign	0.218 benign	0.51 pathogenic
PhyloP	7.49995 conserved	1.58957 conserved	1.48181 conserved	−0.120591 fast-evolving	4.417 conserved
PolyPhen-2	1.0 probably damaging	0.852 possibly damaging	0.999 probably damaging	0.012 benign	1.0 probably damaging
PrimateAI	0.7044 uncertain	0.6351 uncertain	0.5129 tolerated	0.3487 tolerated	0.4389 tolerated
REVEL	0.484 uncertain	0.12 benign	0.104 benign	0.025 benign	0.488 uncertain
SIFT	0.00 deleterious	0.40 tolerated	0.16 tolerated	0.44 tolerated	0.01 deleterious
VEST4	0.566, *p* = 0.146 pathogenic	0.363, *p* = 0.271 benign	0.426, *p* = 0.225 benign	0.175, *p* = 0.58 benign	0.356, *p* = 0.279 benign

## Data Availability

The data presented in this study are available on request from the corresponding author. The data are not publicly available due to patient privacy considerations.

## Supplementary Materials

The supporting information can be downloaded at https://www.mdpi.com/article/10.3390/ijms26136535/s1.
