# Supplementary material for: Genetic Variants in the Extracellular Matrix Gene TNXB Predicted to Alter Fibronectin III Domains in Arterial Aneurysmal and Dissection Diseases
Source: Int J Mol Sci. 2025 Jul 7;26(13):6535. doi: 10.3390/ijms26136535 (PMC12250116; doi:10.3390/ijms26136535)
Supplement: Supplementary file 1 [file ijms-26-06535-s001.zip › ijms-3533594-supplementary.pdf]

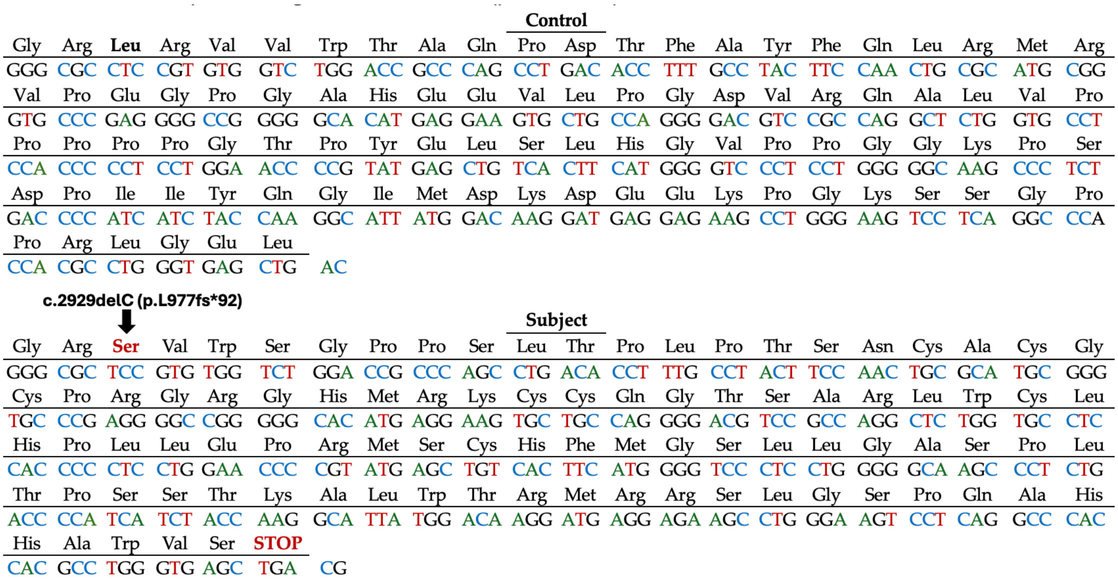

Scheme 1. Variant and control sequence illustrating 92 amino acids altered by p.Leu977fs*92.

Table S1. Genetic results for patients with arterial aneurysms and variants in the TNXB gene. Domain location was based on updated positional data from UniProt [68]. Allele frequency of each variant was obtained from gnomAD [18]. Associated phenotypes and pathogenicity classifications were reported in ClinVar [24]. Abbreviations: CV, Cardiovascular; VR, Vesicoureteral Reflux; SLE, Systemic Lupus Erythematosus; EDS, Ehlers-Danlos Syndrome; US, Uncertain Significance; LB, Likely Benign; (#) indicates number of submissions associated with that classification; gnomAD frequency represents (total allele count / total allele frequency).

Subject	Variant	Variant Type	Affected Domain	Allele Frequency	Associated Phenotypes	Pathogenicity
1	p.L977fs*92<br>c.2929delC	Frameshift	Truncation at FNIII-3	0.0003109%<br>5 / 1608118	CV	Pathogenic (1)
2	p.P2837fs*19<br>c.8510_8511delCT	Frameshift	Disordered Region	0.006199%<br>1 / 1613288	Unknown<br>CV	Pathogenic (1)
3	p.D1512_V1517del<br>c.4535_4552del18	In-frame deletion	Truncation at FNIII-8	0.01141%<br>184 / 1612820	CV, VR, SLE	Uncertain Significance (2)
4	p.G603D<br>c.1808G>A	Missense	EGF-15	0.003162%<br>51 / 1613108	EDS, CV	Uncertain Significance (3)
5	p.S928Y<br>c.2783C>A	Missense	FNIII-2	0.08805%<br>1335 / 1516236	EDS (2), VR, CV	Uncertain Significance (7)
6	p.T982I<br>c.2945C>T	Missense	FNIII-3	0.02338%<br>376 / 1608292	CV	Conflicting<br>US(1), LB(1)
7, 8	p.S1830R<br>c.5488A>C	Missense	FNIII-11	0.0969%<br>1563 / 1612968	EDS(2), CV, VR	Conflicting<br>US(3), LB(4)
9	p.S2751L<br>c.8252C>T	Missense	FNIII-20	0.00003224%<br>52 / 1612840	CV, EDS, VR	Uncertain Significance (2)

Table S2. The 35-gene panel and their respective NCBI (National Center for Biotechnology Information) sequences investigated at our program in patients with aneurysmal diseases [67].

Gene	Sequence	Gene	Sequence	Gene	Sequence
ACTA2	NM_001613.2	*FKBP14*	NM_017946.2	*PRKG1*	NM_006258.3
BGN	NM_001711.4	*FLNA*	NM_001456.3	*SKI*	NM_003036.3
CBS	NM_000071.2	*FOXE3*	NM_012186.2	*SLC2A10*	NM_030777.3
CHST14	NM_130468.3	*LOX*	NM_002317.5	*SMAD3*	NM_005902.3
COL1A1	NM_000088.3	*MAT2A*	NM_005911.5	*SMAD4*	NM_005359.5
COL1A2	NM_000089.3	*MED12*	NM_005120.2	*TGFB2*	NM_003238.3
COL3A1	NM_000090.3	*MFAP5*	NM_003480.2	*TGFB3*	NM_003239.2
COL5A1	NM_000093.4	*MYH11*	NM_002474.2	*TGFBR1*	NM_004612.2
COL5A2	NM_000393.3	*MYLK*	NM_053025.3	*TGFBR2*	NM_003242.5
EFEMP2	NM_016938.4	*NOTCH1*	NM_017617.3	*TNXB*	NM_019105.6
FBN1	NM_000138.4	*PLOD1*	NM_000302.3	*ZNF469*	NM_001127464.1
FBN2	NM_001999.3	*PRDM5*	NM_018699.2		

Table S3. Global pLDDT of each structure prediction obtained from AlphaFold 3 server with accompanying sequence length and reference data.

Variant	Reference Sequence or AFDB Fragment	Sequence Length	Global pLDDT
p.L977fs*92	NM_001365276.2	1067	68.71
p.P2837fs*19	NM_019105.8	2854	70.03
p.D1512_V1517del	P22105-F5	1394	78.66
p.G603D	P22105-F2	1400	79.59
p.S928Y	P22105-F2	1400	80.20
p.T982I	P22105-F2	1400	79.63
p.S1830R	P22105-F6	1400	79.68
p.S2751L	P22105-F11	1400	79.36
